# Craniofacial Reconstruction Method Based on Region Fusion Strategy

**DOI:** 10.1155/2020/8835179

**Published:** 2020-12-04

**Authors:** Yang Wen, Zhou Mingquan, Lin Pengyue, Geng Guohua, Liu Xiaoning, Li Kang

**Affiliations:** College of Information Science and Technology, Northwest University, Xi'an, China

## Abstract

Craniofacial reconstruction is to estimate a person's face model from the skull. It can be applied in many fields such as forensic medicine, archaeology, and face animation. Craniofacial reconstruction is based on the relationship between the skull and the face to reconstruct the facial appearance from the skull. However, the craniofacial structure is very complex and the relationship is not the same in different craniofacial regions. To better represent the shape changes of the skull and face and make better use of the correlation between different local regions, a new craniofacial reconstruction method based on region fusion strategy is proposed in this paper. This method has the flexibility of finding the nonlinear relationship between skull and face variables and is easy to solve. Firstly, the skull and face are divided into five corresponding local regions; secondly, the five regions of skull and face are mapped to low-dimensional latent space using Gaussian process latent variable model (GP-LVM), and the nonlinear features between skull and face are extracted; then, least square support vector regression (LSSVR) model is trained in latent space to establish the mapping relationship between skull region and face region; finally, perform regional fusion to achieve overall reconstruction. For the unknown skull, first divide the region, then project it into the latent space of the skull region, then use the trained LSSVR model to reconstruct the face of the corresponding region, and finally perform regional fusion to realize the face reconstruction of the unknown skull. The experimental results show that the method is effective. Compared with other regression methods, our method is optimal. In addition, we add attributes such as age and body mass index (BMI) to the mappings to achieve face reconstruction with different attributes.

## 1. Introduction

The goal of Craniofacial Reconstruction (CFR) [1] is to estimate the facial outlook of an individual according to the skull. The geometric shape of the skull determines the basic shape of the face. Craniofacial reconstruction technology is based on the relationship between skull and face in forensic medicine and anthropology to realize the facial reconstruction of an unknown skull. This technology has been applied in criminal investigation, archaeological anthropology, and other fields.

Craniofacial reconstruction includes traditional manual face reconstruction and computer-aided face reconstruction. Traditional manual craniofacial reconstruction method has a long period of facial reconstruction, the results are easily affected by subjective factors, and the reconstruction personnel needs to have an anatomical and artistic basis. Because the traditional manual method has measurement errors when measuring the thickness of soft tissue by acupuncture or knife method and can only measure the thickness of soft tissue at a few characteristic points, the reconstruction result is inaccurate.

The computer-aided facial reconstruction method has the advantages of fast execution, objective reconstruction results, and easy editing, and it can accurately calculate the soft tissue thickness of large living sample data sets through computer technology. At present, this technology has become the mainstream of facial reconstruction technology. Computer-aided facial reconstruction methods are currently divided into three categories:

1. *Craniofacial Reconstruction Method based on Soft Tissue Thickness*. This type of method first measures the thickness of the soft tissue at the characteristic point, uses it as the thickness of the soft tissue at the corresponding position of the skull to be reconstructed, and then realizes the face reconstruction through surface interpolation. Philips and Smuts [2] and Zhou et al. [3] proposed to use CT to obtain living sample data and accurately measure soft tissue data of large data set, so as to ensure the accuracy of facial reconstruction. Archer [4] firstly measured the soft tissue thickness at the feature points and then used the hierarchical B-spline interpolation function to realize the facial reconstruction of the skull to be reconstructed. Shui et al. [5] proposed a craniofacial reconstruction method based on dense FSTT statistical data and CT data. Gietzen et al. [6] presented an automated method based on a parametric skull model, a parametric head model, and a statistic of FSTT for reconstructing the face for a given skull. This type of method only depends on the thickness of the soft tissue at the feature points, and usually the number of feature points is small, and it is difficult to represent a face with rich details, so the reconstruction accuracy of this method is not high
2. *Method of Craniofacial Reconstruction based on Template Deformation*. This method does not need to measure the thickness of soft tissue. Firstly, the nonrigid registration deformation of the reference skull to the reconstructed skull is realized based on the skull feature points, and then the transformation is applied to the reference face model to realize the facial reconstruction. Turner et al. [7] used a thin plate spline function algorithm to achieve skull registration. To ensure the accuracy of registration results, TPS registration was used many times in the registration process. Li et al. [8] used the moving least square method to achieve the registration of the reference facial feature points and the facial feature points of the skull to be reconstructed calculated based on the thickness of soft tissue, so as to realize the facial reconstruction. Deng et al. [9] present a novel skull registration method that can match the two skulls closely, so as to improve the accuracy of the reconstruction. It combines both global and local deformations. Hu et al. [10] proposed an automatic 3D face reconstruction method based on hierarchical dense deformation model. To construct the model, the skull and face samples are acquired by a CT scanner and represented as dense triangle mesh. Then, a nonrigid dense mesh registration algorithm is presented to align all the samples in point-to-point correspondence. Based on the aligned samples, a global deformable model is constructed, and three local models are constructed from the segmented patches of the eye, nose, and mouth. For a given skull, the global and local deformable models are iteratively matched with it, and the reconstructed facial surface is obtained by fusing the global and local reconstruction results. Maya and Chiara [11] present a numerical method for facial reconstruction. This approach combines classical features as the use of a skulls/faces database to learn the relations between the two items and more original aspects: (i) use an original shape matching method to link the unknown skull to the database templates; (ii) the final face is seen as an elastic 3D mask which is adapted onto the unknown skull. Generally, the reconstruction results with this kind of methods may contain some visually unexpected features of the template face. What is worse, great model bias may occur if an inappropriate template is chosen
3. *Method of Craniofacial Reconstruction based on Statistical Model*. The statistical model method is an improvement of the template deformation method. The statistical shape model based on the 3D craniofacial database can mine the potential relationship between the skull and face and effectively eliminate the model error of the single template deformation method. If there are enough data samples, a good reconstruction effect can be obtained. Claes et al. [12] used dense facial points and 52 landmarks to represent the face and skull and used PCA to construct a combined statistical shape model. Berar et al. [13] used principal component analysis to construct a statistical model composed of skull and facial models based on a large number of skull and facial models and used the statistical model to achieve facial reconstruction. Zhang et al. [14] propose a face appearance reconstruction algorithm based on a Regional Statistical Craniofacial model. The shape of the craniofacial model is decomposed into a few segments, such as the eyes, the nose, and the mouth regions; then the joint statistical models of different regions are constructed. Finally, the different regions are assembled together to achieve a completed face model. Some researchers use regression methods to extract the relationship between skull and face. Berar et al. [15] use the Latent Root Regression method to predict the face shape from a set of skull landmarks, where the face is represented as a sparse mesh. The reconstruction is highly affected by the quantity and localization accuracy of the landmarks. Paysan et al. [16] estimate the regression from face to skull by ridge regression technique and find the mappings from faces to some attributes by support vector regression. Using the user-defined attributes as constraints, they optimize an objective function defined by predicted skull error and attribute error. Tilotta et al. [17] proposed an extended normal vector field representation for the chin and nose regions of the skull and established a mapping model between the two normal vector fields using nonparametric regression. Duan et al. [18] proposed a craniofacial reconstruction method based on the regression model, in which a statistical shape model is built for skulls and faces, respectively, and the relationship between them is extracted in the shape parameter spaces through partial least square regression. Craniofacial reconstruction is realized by using the relationship and the face statistical shape model. All these works established the regression model in the linear subspaces, which cannot extract complex nonlinear craniofacial features

Gaussian process latent variable model is an effective method for nonlinear mapping of high-dimensional data [19, 20]. LSSVR [21] was proposed by Suykens and Vandewalle in 1999. Compared with the linear modeling method, LSSVR has more advantages in solving nonlinear problems. In addition, due to the complexity of craniofacial structure, the relationship between skull and face is different in different craniofacial regions. Some studies reveal that the local shape model is better than the global model to represent local shape variety [17, 22]. Therefore, we divide the skull and face into five regions and then learn the mapping relationship of each region independently. For the unknown skull, five face regions are obtained through the learned five mappings, and finally, the region fusion is performed to achieve face reconstruction. This paper proposes a craniofacial reconstruction method based on regional fusion strategy. Firstly, the skull and face are divided into five corresponding local feature regions. Then, the five regions of the skull and face are mapped to low-dimensional latent space using the Gaussian process latent variable model, and the LSSVR model is trained in the latent space, that is, the five maps of skull region to corresponding facial region are established. For the unknown skull, we divided it into five regions and projected it into the latent space of the skull, and used the LSSVR model to reconstruct the face of the corresponding region. Finally, perform regional fusion to achieve overall reconstruction.

The contributions of this paper are as follows: (1) both the Gaussian process latent variable model and LSSVR have more advantages in solving nonlinear problems. They can effectively extract the complex nonlinear relationship between skull and facial morphology and better represent the changes of craniofacial morphology. (2) To represent the craniofacial shape changes well, we established the LSSVR model of five regions and reconstructed the face through the regional fusion strategy, thus provide a more face-like facial approximation for the unknown skull. (3) The proposed method has little manual intervention, which can discard the noise and error that manual operations introduce.

## 2. Material

**Figure 1:**
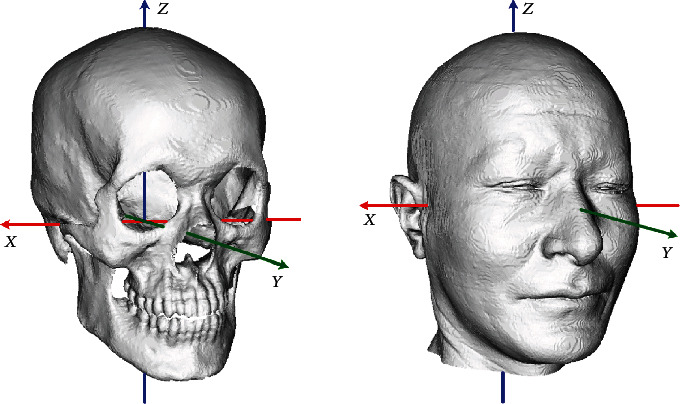
The skull and face of a sample in the uniform coordinate system.

Our research has been approved by the ethics committee of the Northwest University of China. The study was carried out on a database of 200 whole head CT scans on voluntary persons that mostly come from the Han ethnic group in North of China, age 19–75 years for females and 21–67 years for males. There are 90 females and 110 males. The CT images were obtained by a clinical multislice CT scanner system (Siemens Sensation 16) in the Xianyang hospital located in western China. The images of each subject are restored in DICOM format with a size of approximately 512 × 512 × 250. The original CT slice images are processed by feature detection method to extract skull and face borders. The 3D skull and skin surfaces are reconstructed by a marching cubes algorithm [23] and represented as triangle meshes including about 150000 and 220000 vertices, respectively. All the heads are substantially complete. In detail, each skull contains all the bones from calvarias to jaw and has the full mouth of teeth, and each face has no missing part either. In addition, the subject's properties for each head such as age, gender, and BMI are stored also. To eliminate the inconsistency caused by position, attitude, scale, and other factors during data acquisition, all samples are converted to the unified Frankfurt coordinate system and normalized [24], as shown in Figure 1.

**Figure 2:**
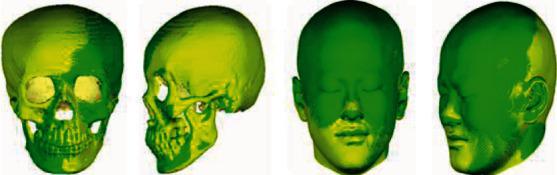
The results of skull and face registration.

The original skull and face mesh have different connections under different number of vertices. To eliminate the influence of data factors on the establishment of latent space, it is necessary to establish dense point correspondence between training samples, that is, data registration. Due to the nonrigid deformation of a complex curved surface, it is a challenging problem to establish point correspondence for skulls and faces. To establish a dense point correspondence between each skull (face) and a reference skull (face) in our database, we used the 3D surface registration method [25] introduced by other researchers in our cooperation team. First, the global deformation based on the general thin-plate spline is used to roughly align the two skulls (face skin). After the global deformation, the local mismatched areas need to be adjusted. Use the deformation based on the radial basis function to adjust the local regions and repeat this operation until the maximum error between the two skulls (face) meets the threshold condition or reaches the set number of iterations. Through the experiment, the skull after 5 times of local deformation will establish a good point corresponding relationship; face after 3 times of local deformation will establish a good point corresponding relationship. The registration results of the skull and face are shown in Figure 2.

## 3. Method

**Figure 3:**
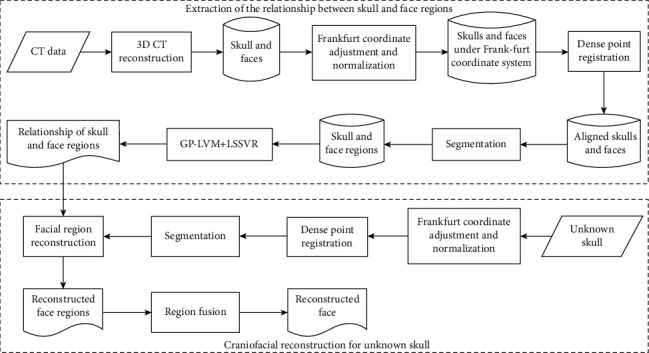
The framework of our method.

The framework of the method in this paper is shown in Figure 3.

### 3.1. The Skull and Face Were Divided into Regions

We divided the skull and face into five regions, namely, left eye, right eye, nose, mouth, and frame region. In this work, we use the method of craniofacial partition based on fuzzy *c*-means clustering [26]. Firstly, robust principal component analysis [27] is used to extract feature vectors, and then fuzzy *c*-means clustering is used to partition the craniofacial model. Finally, similar regions are combined with collaborative segmentation to reduce the number of regions.

Let *x*_*j*_ be the eigenvector of the craniofacial model after dimensionality reduction by robust principal component analysis corresponding to the *j*-th vertex in the craniofacial data, *v*_*i*_ is the eigenvector of the *i*-th cluster center, *C* is the number of craniofacial regions, and *N* is the number of craniofacial vertices. The objective function can be expressed as follows:

(1) $$\begin{array}{c} \min\;\;J=\overset{N}{\underset{j=1}{\sum }}\overset{C}{\underset{i=1}{\sum }}{u_{ij}}^{m}{\left\|v_{i}-x_{j}\right\|}^{2}\\ \begin{array}{cc} \text{s}.\text{t}. & \overset{C}{\underset{i=1}{\sum }}u_{ij}=1,u_{ij}\in \left[0,1\right],i=1,2\cdots C,j=1,2,\cdots N \end{array}, \end{array}$$

where *u*_*ij*_ represents the degree to which *x*_*j*_ belongs to the regional category, and *m* > 1 represents the weighted index, that is, the degree of fuzziness. The objective function can be converted using the Lagrangian multiplier method to:

(2) $$\begin{array}{c} L_{m}=\overset{N}{\underset{j=1}{\sum }}\left(\overset{C}{\underset{i=1}{\sum }}{u_{ij}}^{m}{\left\|v_{i}-x_{j}\right\|}^{2}+\lambda \left(1-\overset{C}{\underset{i=1}{\sum }}u_{ij}\right)\right). \end{array}$$

Optimize the objective function *L*_*m*_, take the partial derivatives of *u*_*ij*_, *v*_*i*_, and *λ*, and set the partial derivative result to 0 to obtain the iterative formula of the cluster center *v*_*i*_ and the membership degree *u*_*ij*_, as shown in the following formula (3):

(3) $$\begin{array}{c} v_{i}=\frac{\sum_{k=1}^{N}u_{ik}^{m}x_{k}}{\sum_{k=1}^{N}u_{ik}^{m}}, \end{array}$$

(4) $$\begin{array}{c} u_{ij}=\frac{{\left(v_{i}-x_{j}\right)}^{2/m-1}}{\sum_{k=1}^{C}{\left(v_{k}-x_{j}\right)}^{2/m-1}}. \end{array}$$

After introducing the Lagrange multiplier, the optimization process is transformed into an iterative process of alternately updating the cluster center *v*_*i*_ and the membership matrix *u*. When *u*^*t*^ − *u*^*t*+1^ < *ε* or the preset number of iterations is reached, the clustering algorithm stops iterative updates. After stopping the iterative update, the optimal membership matrix is obtained, the intraclass reaches a high degree of aggregation, and the class reaches a high degree of discrimination. According to the degree of membership of each vertex of the cranial surface relative to each category, the category to which each vertex belongs is obtained:

(5) $$\begin{array}{c} u_{ik}=\max\left(u_{1k},u_{2k},\cdots ,u_{ck}\right). \end{array}$$

**Figure 4:**
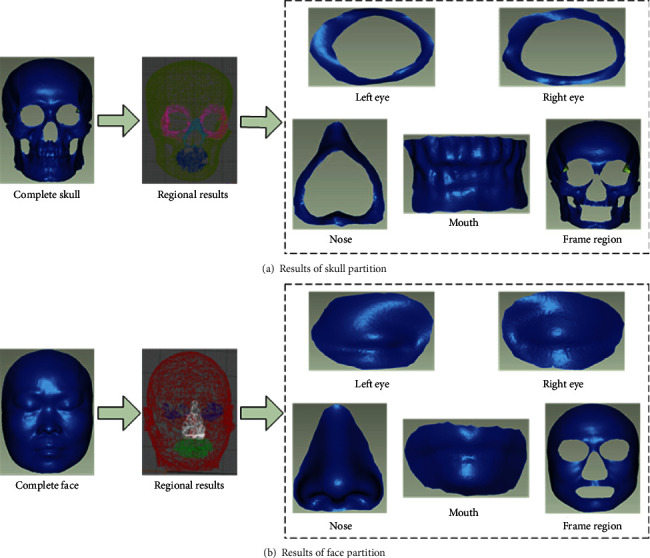
Results of various regions of the skull and face.

On the basis of the whole model, the craniofacial region is divided by the fuzzy *c*-means clustering algorithm. In the process of partitioning, the fuzzy weighting index and the number of clusters need to be set. Based on experience, we choose the fuzzy weighting index *m* = 2 and the number of clusters *C* = 5. The division results of the skull and face are shown in Figure 4.

### 3.2. Gaussian Process Latent Variable Model

The Gaussian process latent variable model is a dimensionality reduction algorithm using the Gaussian process. The Gaussian process is a set of arbitrary finite random variables, and the variables of the set should satisfy Gaussian distribution. Gaussian process is determined by mean function and kernel function.

(6) $$\begin{array}{c} f\left(x\right)∼\text{GP}\left[m_{\left(x\right)},k\left(x,x^{\prime }\right)\right]. \end{array}$$

The model *y* = *f*(*x*) + *δ*^2^, where *x* is the input, *y* is the output, *δ*^2^ ~ *N*(0, *σ*_*n*_^2^).

The posterior distribution of *y*_∗_ in training set *y* and test point *x*_∗_ is

(7) $$\begin{array}{c} P\left(y_{*}\;|\;X,y,x_{*}\right)∼N\left(\mu_{*},\Sigma_{*}\right), \end{array}$$

(8) $$\begin{array}{c} \mu_{*}=k\left(x_{*},K\right){\left[k\left(X,X\right)+\sigma_{n}^{2}I_{n}\right]}^{-1}y, \end{array}$$

(9) $$\begin{array}{c} \Sigma_{*}=k\left(x_{*},x_{*}\right)-k\left(x_{*},X\right){\left[k\left(X,X\right)+\sigma_{n}^{2}I_{n}\right]}^{-1}k\left(X,x_{*}\right), \end{array}$$

where *μ*_∗_ is the mean value, *Σ*_∗_ is the variance, *I*_*n*_ is the *n*-dimensional identity matrix, and **k**(*X*, *X*) is the symmetric covariance matrix.

To achieve the optimal training effect, the square exponential covariance function is used as the kernel function of Gaussian process regression

(10) $$\begin{array}{c} k\left(x,x^{\prime }\right)=\sigma_{f}^{2}\exp\left[-\frac{1}{2}\left(x-x^{\prime }\right)M^{-1}\left(x-x^{\prime }\right)\right], \end{array}$$

where *θ* = {*M*, *σ*_*n*_^2^, *σ*_*f*_^2^} is the hyperparameter, the maximum likelihood estimation function is used to optimize the Gaussian hyper-parameter, as shown in the following equation (11),

(11) $$\begin{array}{c} L=\log P\left(y\;|\;x,\theta \right)=\frac{1}{2}\log\left[\det\left(k+\sigma_{n}^{2}I_{n}\right)\right]-\frac{1}{2}y^{T}{\left[k+\sigma_{n}^{2}I_{n}\right]}^{-1}y-\frac{N}{2}\log2\pi . \end{array}$$

The partial derivative of the likelihood function *L* is calculated to obtain the optimal super parameter {*M*, *σ*_*n*_^2^, *σ*_*f*_^2^}. The predicted mean value and variance can be obtained by substituting it into equation (6).

### 3.3. The Latent Space Representation of Skull and Face

To reduce the data dimension and simplify the calculation, we first use GP-LVM to represent the skull and face data in the latent space, that is, to extract the non-linear characteristics of the skull and face. The skull data is denoted as **S** = [**s**_1_, **s**_2_, ⋯,**s**_*N*_]^*T*^, and its latent space data is denoted as **S**′ = [**s**_1_′, **s**_2_′, ⋯,**s**_*N*_′]^*T*^; the face data is denoted as **F** = [**f**_1_, **f**_2_, ⋯,**f**_*N*_]^*T*^, and its latent space data is denoted as **F**′ = [**f**_1_′, **f**_2_′, ⋯,**f**_*N*_′]^*T*^. According to the above formula (7), the relationship between the original data and its latent spatial data can be established, as shown in the following formula,

(12) $$\begin{array}{c} \begin{array}{c} P\left(s_{*}^{\prime }\;|\;S,s,s_{*}\right)∼N_{S}\left(\mu_{*},\Sigma_{*}\right),\\ P\left(f_{*}^{\prime }\;|\;F,f,f_{*}\right)∼N_{F}\left(\mu_{*},\Sigma_{*}\right). \end{array} \end{array}$$

The above formula can be solved using the process described in Section 3.2 and gradient-based optimization [28].

### 3.4. Establish the Regression Relationship between Skull and Face by LSSVR Model

Through the above process, the skull and face have been converted to low-dimensional latent space representation. Because of the kernel technology used in support vector regression, it is very suitable for extracting the nonlinear relationship between variables. In this paper, we use the least square support vector regression to establish the mapping relationship between the skull latent spatial data **S**′ = [**s**_1_′, **s**_2_′, ⋯,**s**_*N*_′]^*T*^ and the face latent spatial data **F** = [**f**_1_, **f**_2_, ⋯,**f**_*N*_]^*T*^.

We use the radial basis function as the kernel function. In the training phase, LSSVR is described as an optimization problem,

(13) $$\begin{array}{c} \begin{array}{c} \left[\alpha ,b\right]=\underset{\alpha \in {\mathbb{R}}^{\vartheta },b}{\arg\min}\left[\frac{1}{2}{\left\|\omega \right\|}^{2}+C\overset{\vartheta }{\underset{i=1}{\sum }}\xi_{i}\right]\\ s.t.p_{yi}-\left(\overset{\vartheta }{\underset{i=1}{\sum }}\alpha_{i}\exp\left(\frac{{\left\|\omega -s_{i}\right\|}^{2}}{2\sigma^{2}}\right)+b\right)<\xi_{i}^{2}, \end{array} \end{array}$$

where *C* is the regularization parameter, *ξ*_*i*_ is the relaxation variable, **α** is the support value, and *b* is the deviation term.

Using the Lagrangian function to solve the equation, we obtain the optimal model parameters **ω** and *b*. Then, the face of each region of the unknown skull can be obtained through the regression model.

(14) $$\begin{array}{c} f\left(s\right)=\overset{\vartheta }{\underset{i=1}{\sum }}\alpha_{i}\exp\left(\frac{{\left\|\omega -s\right\|}^{2}}{2\sigma^{2}}\right)+b. \end{array}$$

### 3.5. Regional Fusion

To get a complete and smooth reconstruction face, after getting the reconstruction face of each region, the next work is to fuse the reconstructed region. Here, the process of regional fusion is realized through the following two steps.

**Step 1 .**

The relative positioning of the overall model and the regional model in space. For the global grid *F* and the regional grid *F*_sub_, the rotation transformation *R*^∗^ and the translation transformation *T*^∗^ are used to adjust the regional grid to the appropriate position of the global grid, where *R*^∗^ and *T*^∗^ are determined by the average distance between the corresponding points of the regional grid and the global grid, namely,

(15) $$\begin{array}{c} \left(R^{*},T^{*}\right)=\arg\min_{R,T}\sum {RP}_{0}+T-P_{1}, \end{array}$$

where *P*_0_ ∈ *F*_sub_, *P*_1_ ∈ *F*is the corresponding point of *P*_0_. Through the transformation of the above steps, the overall model and the regional model can be well-matched in the spatial position with the geometric shape unchanged. Through step 1, the initial fusion of face can be realized.

**Step 2 .**

Smooth splicing of grids based on corresponding points. According to the boundary points of the region model and the corresponding relationship between the region model and the whole model, the boundary stitching is determined to realize the smooth transition of the mesh from the boundary to both sides. The inconsistency of the boundary is eliminated by grid splicing, and the points on the regional grid and the overall grid near the boundary are deformed to the spatial position using the interpolation method. Through step 2, smooth the initial fused face to obtain the final reconstructed face.

**Figure 5:**
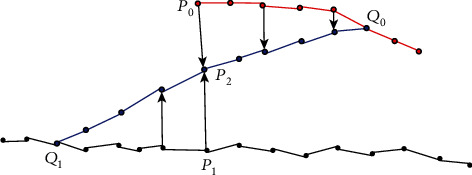
Schematic diagram of the grid interpolation process.

The mesh interpolation process is as follows: for the partition grid boundary *B*_0_ and one point *P*_0_ ∈ *B*_0_, the corresponding boundary on the overall grid (represented by *B*_1_) and the corresponding point *P*_1_ ∈ *B*_1_ of *P*_0_, then calculate the interpolation point *P*_2_ = (*P*_0_ + *P*_1_)/2. Given a scale *S*_0_, the boundary *B*_0_ will shrink to the interior with the step of *S*_0_ to get a contour *B*_0_′ represented by point *Q*_0_, *Q*_0_ ∈ *B*_0_′. *B*_1_ reversely shrinks on the whole mesh and gets the contour *B*_1_′. The mesh interpolation process is shown in Figure 5.

The splicing method is to find a pair of interpolation functions *f*_0_, *f*_1_ which satisfy the following conditions:

(16) $$\begin{array}{c} \left\{\begin{array}{c} f_{0}\left(Q_{0}\right)=Q_{0},Q_{0}\in {B_{0}}^{\prime },\\ f_{0}\left(P_{0}\right)=P_{2},P_{0}\in B_{0},\\ f_{1}\left(Q_{1}\right)=Q_{1},Q_{1}\in {B_{1}}^{\prime },\\ f_{0}\left(P_{1}\right)=P_{2},P_{1}\in B_{1}. \end{array}\right. \end{array}$$

**Figure 6:**
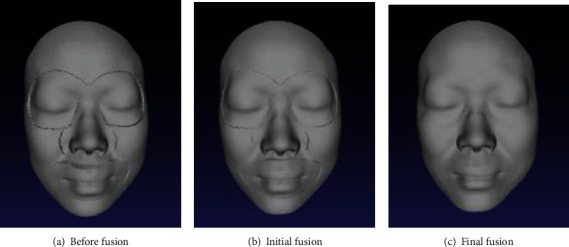
Regional fusion process.

Here, we use the TPS interpolation [29]. According to the properties of TPS transform, the points outside the boundary can realize a smooth transition. After the interpolation function is determined, *f*_0_ can be applied to the points between *B*_0_ and *B*_0_′ on the partition grid, and *f*_1_ can be applied to the points between *B*_1_ and *B*_1_′ on the global grid to obtain the final fusion result. The fusion process is shown in Figure 6.

### 3.6. Craniofacial Reconstruction

For the unknown skull, the method proposed in this paper is used to achieve facial reconstruction through the following steps.

**Step 3 .**

The unknown skull is converted to the Frankfurt coordinate system and normalized, and then the dense point correspondence with the reference skull is established, as described in Section 2.

**Step 4 .**

Use the method described in Section 3.1 to determine five skull regions, which are the left eye, right eye, nose, mouth, and frame region.

**Step 5 .**

The GP-LVM model was used to map the skull regions into the latent space.

**Step 6 .**

The trained LSSVR model was used to reconstruct the face of each region of the unknown skull.

**Step 7 .**

The method described in Section 3.5 is used to fuse the face of the five regions, and the reconstruction of the complete face of the unknown skull is finally realized.

## 4. Experimental Results and Analysis

All of our algorithms were written by us with C++ language, OpenGL, and MATLAB. The database described in Section 2 is divided into a training set and a test set. We randomly select 20 pairs of skull and face data as the test set, and the remaining 180 pairs as the training set. For the LSSVR model, the regularization parameter *C* and RBF kernel parameter *δ*^2^ are optimized by iterative feedback adjustment. The initial *C* is set to 1000, and *δ*^2^ is set to 100. To measure the reconstruction error, we define the average reconstruction error. The average reconstruction error is the average of the distance between all the points on the reconstructed face and their corresponding points on the real face, and the unit of the distance is mm. The average reconstruction error (averageError) is defined as

(17) $$\begin{array}{c} \text{averageError}=\frac{1}{n}\overset{n}{\underset{i=1}{\sum }}\left\|{\text{reconFace}}_{i}-{\text{realFace}}_{i}\right\|, \end{array}$$

where *n* is the number of vertices in the reconstructed face, *i* is the vertex index, reconFace_*i*_ is a vertex in the reconstructed face, realFace_*i*_ is the corresponding vertex in the real face, and ‖·‖ is the Euclidean distance.

### 4.1. Reconstruction Results

**Figure 7:**
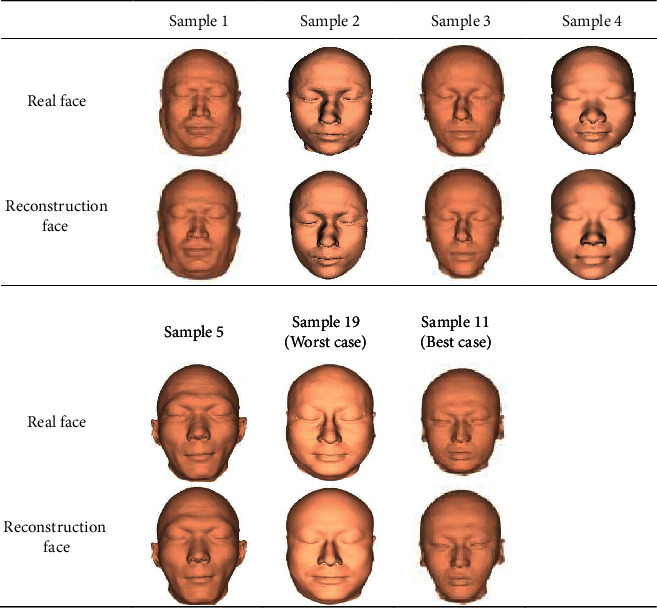
Results of facial reconstruction of some samples.

In this section of the experiment, we use 180 pairs of training samples in the database in Section 2 to establish an LSSVR model, and then verify on 20 pairs of test set samples, and finally compare the reconstructed face with the real face of the test case.  Figure 7 shows the reconstruction results of some of our samples. We also conducted a subjective evaluation of the reconstruction results of the 20 test cases. We recruited 10 volunteers to compare the reconstruction results with the photos of the test cases and gave the evaluation scores. Ten volunteers were numbered with P1~P10. All the volunteers observed the recovery results and photos for the first time, and each volunteer scored independently and was not affected by others. The evaluation score is defined as 1 means very poor, 2 means poor, 3 means up to grade, 4 means good, and 5 means very good. The subjective evaluation scores and reconstruction errors of each test sample are shown in Table 1. We also calculated the minimum reconstruction error, maximum reconstruction error, average reconstruction error, and standard deviation of the five region reconstruction results of test samples, as well as the minimum reconstruction error, maximum reconstruction error, average reconstruction error, and standard deviation of the whole face reconstruction results after regional fusion. The results are shown in Table 2.

Due to the limited space, Figure 7 shows the reconstruction results of five randomly selected samples, the best-case reconstruction results, and the worst-case reconstruction results. As can be seen from Figure 7, from the perspective of visual effect, the reconstructed face is very similar to the real face. Sample 19 is the worst-case reconstruction result, and sample 11 is the best case reconstruction result. From the perspective of visual effect, in the reconstruction results of sample 19, the facial features and the real face are very similar, but the face shape is different; in the reconstruction results of sample 11, the facial features and face shapes are similar to the real face, and the degree of similarity is higher than other test samples. As can be seen from Table 1, there are 16 samples with an evaluation score greater than 3, and 4 samples with an evaluation score greater than 4. Therefore, from the perspective of subjective evaluation, the reconstruction method in this paper is still very effective. Although the subjective assessment method is not scientific enough, it can also reflect the reconstruction effect to a certain extent, because one of the practical uses of craniofacial reconstruction is to identify the skull. In addition, it can be seen that the reconstruction error is 0.61 mm in the best case and 2.32 mm in the worst case. It can be seen from the error results of the five regions and the complete reconstruction face in Table 2 and that the eye region has the smallest error and the frame region has the largest error, which is consistent with the research conclusion of Deng et al. [25]. In addition, the average reconstruction error of the method in this paper is 1.49 mm, which is smaller than the average reconstruction error of the other four regions except for the frame region, which indicates that the frame region reconstruction results have a bad impact on the overall reconstruction results.

### 4.2. Comparison of Global and Local-Based Reconstruction Methods

To verify the superiority of the region-based craniofacial reconstruction method proposed in this paper, we compared with the global reconstruction method. We call the method in this paper a local craniofacial reconstruction method. We describe a global-based craniofacial reconstruction method as follows: the method proposed in this paper is used, but the data of the skull and face are not divided into regions. The skull and face are directly used as objects, and the GP-LVM is used to map the skull and face to the low-dimensional latent space, respectively. The LSSVR model is trained in the latent space to establish the mapping relationship between the whole skull and the whole face, and then the trained LSSVR model is used to reconstruct the face for the unknown skull. The LSSVR model is trained on the training set by using the global reconstruction method and the local-based reconstruction method, respectively. The parameters of the training stage are the same, and then the test is carried out on the test set.

**Figure 8:**
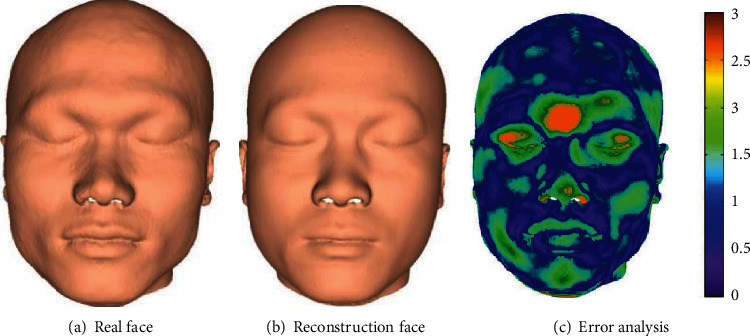
Global-based reconstruction method.

**Figure 9:**
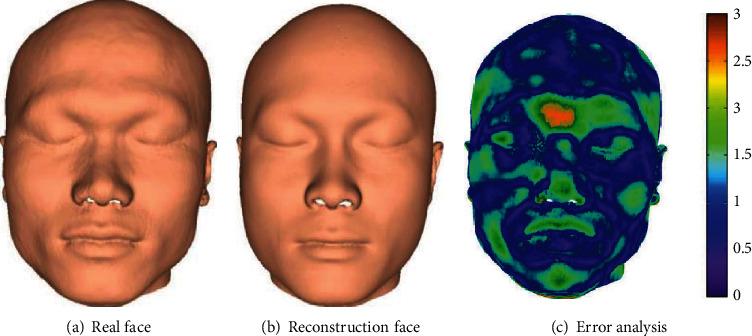
Local-based reconstruction method (our method).

**Figure 10:**
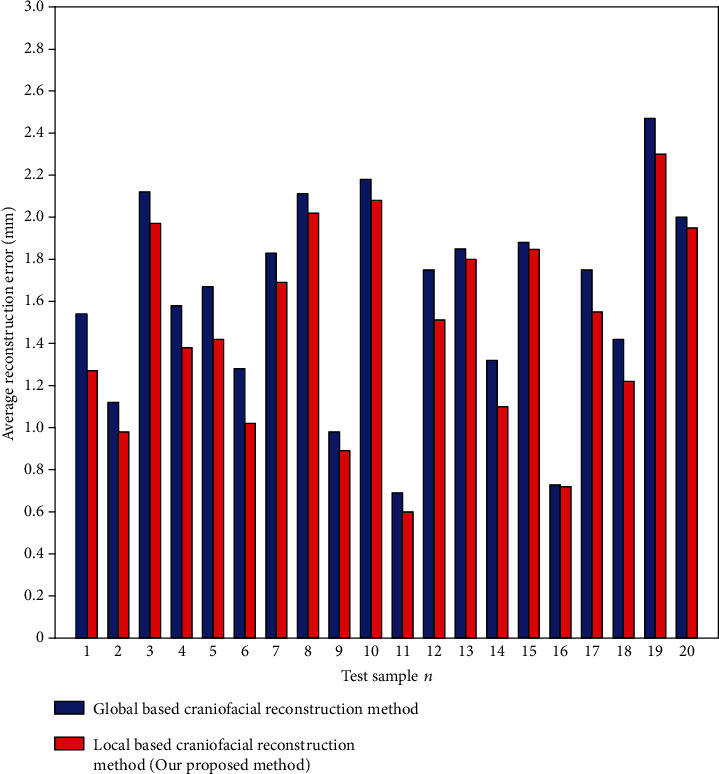
The average reconstruction error of the local and global craniofacial reconstruction method on 20 test samples was analyzed.

Figures 8 and 9 show the results of the global reconstruction method and the local reconstruction method, respectively.  Figure 10 shows a comparison of the average reconstruction errors of the two methods on the test samples. From the visual point of view, it can be seen from Figures 8(b) and 9(b) that the reconstruction results of the two methods are good, which is close to the real face. From the error analysis diagrams in Figures 8(c) and 9(c), it can be seen that the proposed method is superior to the global-based craniofacial reconstruction method. A rainbow is used to show the difference between each corresponding point. Purple represents minimum distance, and yellow represents maximal distance. From the quantitative point of view, it can be seen from Figure 10 that for most (almost all) samples, the average reconstruction error of the proposed method is smaller than that of the global craniofacial reconstruction method. On all test samples, the average reconstruction error of the proposed method is 1.49 mm, while that of the global craniofacial reconstruction method is 1.63 mm. Therefore, from the perspective of quantitative analysis, the method in this paper is better than the global-based craniofacial reconstruction method. This also shows that compared with the whole method, the regional method is more suitable for craniofacial reconstruction, because the human craniofacial structure is very complicated, and the relationship between the skull and the face is different in different craniofacial regions. Regional analysis of skull and face can achieve a more accurate internal relationship between skull and face.

### 4.3. Comparative Analysis of Different Craniofacial Reconstruction Methods

**Figure 11:**
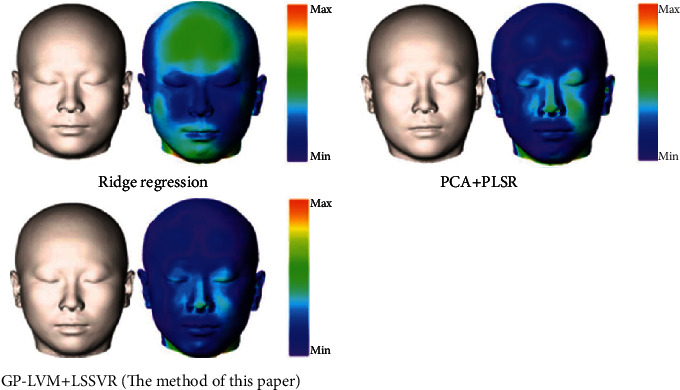
Comparison of the results of the three reconstruction methods.

**Figure 12:**
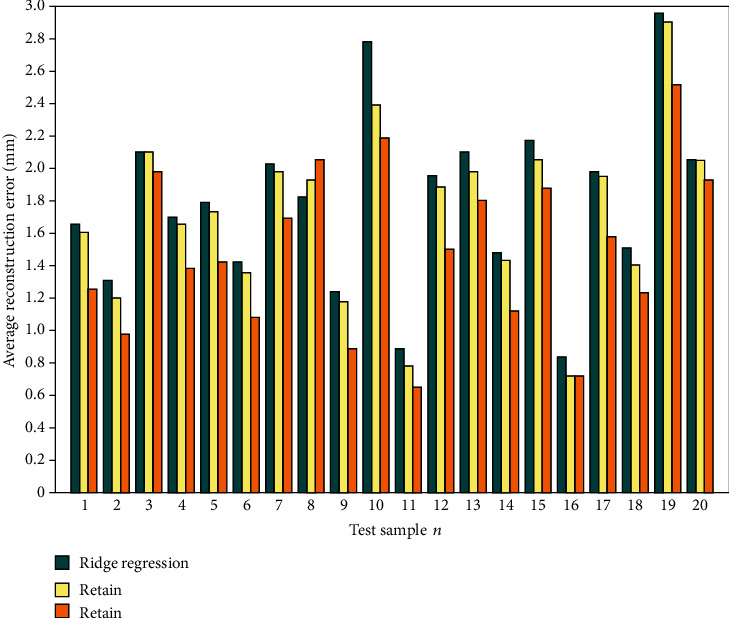
Average reconstruction error of different methods on the test sample set.

To compare with other regression methods, we compare the method in this paper (referred to as GP-LVM+LSSVR) with the ridge regression method [16] and the method of Li et al. [30] (referred to as PCA+PLSR). Ridge regression is a regularized least square regression. When multicollinearity exists between data variables, the general least square regression becomes an ill-posed problem, while ridge regression can solve the multicollinearity problem by adding penalty terms. PLSR is a relatively new linear regression modeling method, which integrates the functions of PCA, multiple linear regression, and canonical correlation analysis to obtain more effective prediction results. We use the same setting for three regression methods in the comparison, that is, both the data and the procedure are the same for two methods. Ridge regression, PCA+PLSR, and GP-LVM+LSSVR were used to reconstruct the face of 20 test cases in the test set, and the average reconstruction error of each sample was calculated.  Figure 11 shows the visual reconstruction error of the reconstruction results of the three methods.  Figure 12 shows the average error comparison of the three methods on the test set sample. In addition, we also count the minimum error, maximum error, and standard deviation of the three methods on the test set, as shown in Table 3.

It can be seen from Figures 11 and 12 that this method is superior to the other two methods in both visual and quantitative analysis. It can be seen from Figure 11 that this method is significantly better than the other two methods in local regions such as cheeks, mouth, and forehead, and from the entire face, it can be seen that the area of the smaller error region of this method is also smaller than the other two methods. As can be seen from Figure 12 that for most samples, the reconstruction error of this method is smaller than the other two methods, and the reconstruction error of PCA+PLSR is smaller than that of the ridge regression method. As can be seen from Table 3 that the minimum error, maximum error, average error, and standard deviation of the ridge regression method are 0.83 mm, 2.96 mm, 1.80 mm, and 1.37 mm, respectively; the minimum error, maximum error, average error, and standard deviation of the PCA+PLSR method are 0.74 mm, 2.89 mm, 1.72 mm, and 1.39 mm, respectively; the minimum error, maximum error, average error, and standard deviation of the GP-LVM+LSSVR method are 0.61 mm, 2.32 mm, 1.49 mm, and 1.14 mm, respectively. The results of the quantitative analysis also show that this method is better than the other two methods; the PCA+PLSR method is better than the ridge regression method.

Because the GP-LVM proposed in this paper extracts the high-order nonlinear features between the shape of the skull and face, it can describe the correlation between skull and face more accurately. On the other hand, due to the kernel technology used in SVR, it is very suitable for extracting the nonlinear relationship between variables. The other two methods are to extract the linear features of the skull and face through principal component analysis and then establish a linear regression relationship for craniofacial reconstruction; this linear mappings method cannot well reflect the complex essential relationship between skull and face. Based on the above analysis, the advantages of GP-LVM and LSSVR enable the proposed method to model the relationship between skull and skin more accurately. Therefore, this method is more effective.

### 4.4. Effects of Age and BMI on Reconstruction Results

**Figure 13:**
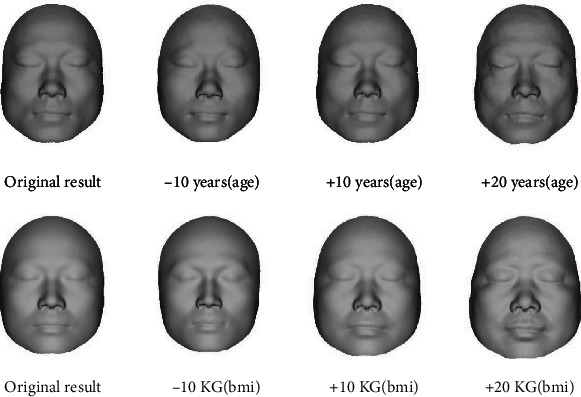
The reconstruction results with variation of attributes.

Because the thickness of human soft tissue changes with the changes of BMI and age, the face will also be affected by these attributes. Therefore, we added BMI and age into the regression model to analyze the influence of attributes on the reconstruction results.  Figure 13 shows the reconstruction results of the sample with BMI and age. The first row is the reconstruction result with age, and the second row is the reconstruction result with BMI. Since BMI is calculated from weight and height, the change of BMI can be controlled by directly changing weight. As can be seen from Figure 13, as BMI and age increase or decrease, the face can become fatter or thinner, older or younger. It indicates that variations of the reconstruction result are approximately rational. This shows that attribute factors will affect the results of craniofacial reconstruction. When conducting craniofacial reconstruction, researchers should appropriately consider the influence of attributes such as BMI and age on the reconstruction results.

## 5. Discussion and Conclusion

The goal of ridge regression is to get a stable estimate by regularizing the least square problem, while PLSR is to extract the most relevant latent variables from skull and face and then establish the mapping relationship through regression modeling. Therefore, PLSR is more effective for the small sample problem of craniofacial reconstruction. PCA+PLSR method is to extract the linear characteristics of the skull and skin through principal component analysis and establish a regression model. This method reflects the global change of the data set and does not represent the local morphological changes of the craniofacial region; the PLSR linear method is used for modeling, which is not sufficient to represent the nonlinear craniofacial deformation. In this paper, the nonlinear features of the skull and skin are extracted in different regions, and the least square support vector regression (LSSVR) is also used to establish the nonlinear relationship between variables. Therefore, the same conclusion can be obtained from the analysis of experimental results and methods, that is, this method is better than the PCA+PLSR method, and the PCA+PLSR method is better than the ridge regression method.

This paper designs four groups of experiments from different angles. The experimental results prove the effectiveness of our method, and four conclusions can be drawn. Firstly, the nonlinear modeling method can obtain a more accurate correlation between skull and face. Secondly, the reconstruction result of the regional method is better than that of the whole method. Third, consideration of properties can improve the craniofacial reconstructions. Last but not least, it is reasonable that the subjective evaluation method is used to evaluate the results of craniofacial reconstruction. The results are basically consistent with the results of the quantitative analysis. Usually, the error of two faces with similar visual effects is also small in quantitative analysis.

Craniofacial reconstruction is the last useful tool to identify the unknown corpse if no other evidence is available in forensic investigation. This paper proposes a new method of craniofacial reconstruction based on regional fusion. The method first divides the skull and face into five regions, namely, the left eye, right eye, nose, mouth, and frame region. Then, the high-order nonlinear features of the skull and face are extracted by GP-LVM, and the mapping relationship between skull and skin is established by LSSVR in the latent space of the skull and face. Finally, this relationship was used to reconstruct the face of the unknown skull. We designed four groups of experiments. The first group evaluated the reconstruction results of the proposed method from both subjective and objective aspects. In the second group of experiments, we compare the proposed method with the whole method, the results show that the reconstruction effect of this method is better, and the reconstruction error is smaller. In the third group of experiments, we compare this method with other regression methods, and the results show that this method is better than the other two methods. In the fourth group of experiments, we analyzed the influence of attributes on the reconstruction results. With the change of BMI, the face will become fat or thin; and with the change of age, the face will become older or younger. The experimental results demonstrate that the local regional shape correlation modeling approach is a more scientific and effective modeling approach in describing craniofacial shape correlation and our reconstruction method can significantly improve the craniofacial reconstruction accuracy.

Some extensions can be proposed for further improvement. Firstly, according to the anatomic knowledge and craniofacial morphology, a more precise and reasonable region definition and segmentation method is proposed. Anatomic landmarks are extracted from the skull and face automatically, which makes the whole process of regional division automatic. In addition, it is necessary to continue to search for the feature attributes that can effectively represent the feature regions of the craniofacial model, mine the dependency relationship between the skull and the face, and divide the skull and the face into more precise regions according to the human physiological structure. At the same time, the ear should be considered as a local area, which will make the craniofacial reconstruction result more complete. Secondly, texture information is added in the reconstruction process to make the result more realistic and reliable. For example, skin color information, hair information, and even eye color information can be added to the reconstructed face.

## Figures and Tables

**Table 1:** Subjective assessment scores of all test samples.

Test sample set	*P*_1_	*P*_2_	*P*_3_	*P*_4_	*P*_5_	*P*_6_	*P*_7_	*P*_8_	*P*_9_	*P*_10_	Average score	Reconstruction error (mm)
Test sample #1	3	3	4	4	4	3	4	4	3	4	3.6	1.29
Test sample #2	4	4	4	5	3	4	5	4	4	3	4.0	1.19
Test sample #3	2	3	3	4	3	2	3	3	1	2	2.6	2.05
Test sample #4	3	3	4	3	5	4	4	3	3	4	3.6	1.30
Test sample #5	4	3	4	3	4	3	3	4	4	4	3.6	1.32
Test sample #6	4	4	3	3	3	5	5	5	3	4	3.9	1.23
Test sample #7	3	4	2	3	4	3	4	3	4	3	3.3	1.55
Test sample #8	2	4	3	1	2	3	3	2	3	3	2.6	2.19
Test sample #9	4	5	3	5	4	4	5	3	4	5	4.2	1.15
Test sample #10	2	2	1	3	3	2	2	1	3	4	2.3	2.22
Test sample #11	5	5	4	5	5	4	4	4	4	4	4.4	0.61
Test sample #12	4	4	4	3	3	4	4	4	3	3	3.6	1.34
Test sample #13	4	3	3	2	3	3	4	3	3	4	3.2	1.57
Test sample #14	5	4	3	3	4	4	4	4	4	3	3.8	1.27
Test sample #15	3	3	4	3	4	4	2	3	3	3	3.2	1.65
Test sample #16	4	4	5	4	4	5	4	5	4	4	4.3	1.02
Test sample #17	3	3	3	4	3	4	3	4	4	4	3.5	1.43
Test sample #18	3	3	4	4	4	5	3	3	5	3	3.7	1.29
Test sample #19	2	2	3	3	1	1	2	2	1	2	1.9	2.32
Test sample #20	4	3	3	3	3	3	2	3	4	4	3.2	1.81

**Table 2:** Average reconstruction error results of test samples.

Region	Min error (mm)	Max error (mm)	Mean error (mm)	S.D. of error (mm)
Left eye	0.54	1.71	1.12	0.73
Right eye	0.56	1.72	1.04	0.66
Nose	0.42	1.59	0.90	0.59
Mouth	0.60	1.93	1.24	0.75
Frame	0.78	3.36	2.05	1.26
Face after regional fusion	0.61	2.32	1.49	1.14

**Table 3:** Reconstruction error comparison of Ridge regression, PCA+PLSR, and GP-LVM+LSSVR.

Methods	Min error (mm)	Max error (mm)	Mean error (mm)	S.D. of error (mm)
Ridge regression	0.83	2.96	1.80	1.37
PCA+PLSR	0.74	2.89	1.72	1.39
GP-LVM+LSSVR	0.61	2.32	1.49	1.14

## Data Availability

The .obj format 3D model data used to support the findings of this study may be released upon application to the Northwest University Visual Technology Institute via the email ghgeng@nwu.edu.cn.
